# Supplementary material for: Nurse–physician collaboration, autonomy, and decision-making ability among intensive care unit nurses in Tehran, Iran: A cross-sectional study
Source: PLoS One. 2025 Dec 9;20(12):e0336430. doi: 10.1371/journal.pone.0336430 (PMC12688092; doi:10.1371/journal.pone.0336430)
Supplement: S2 Appendix — (DOCX) [file pone.0336430.s002.docx]

**
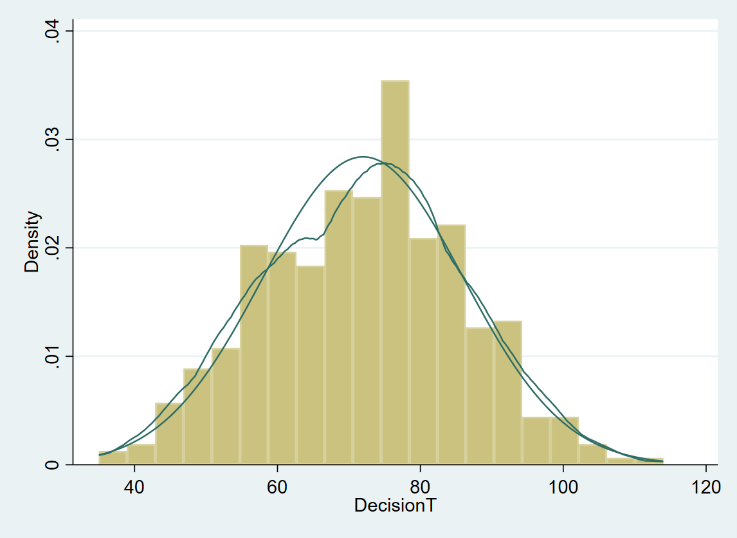

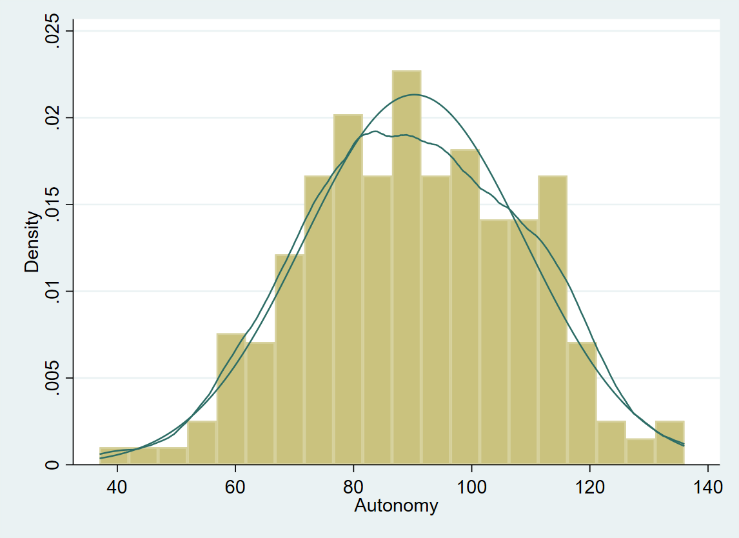
S2 Appendix.** Graphical representations (histogram and Q-Q plot) for checking the normality assumption and results of normality tests.

1. **
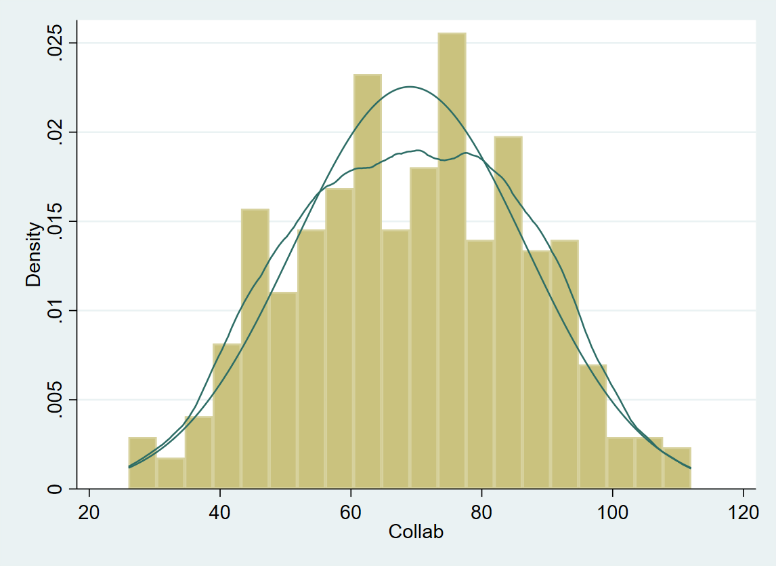
Histogram**
2. **Decision-making 2. Autonomy**

**3.** **Collaboration**

1.
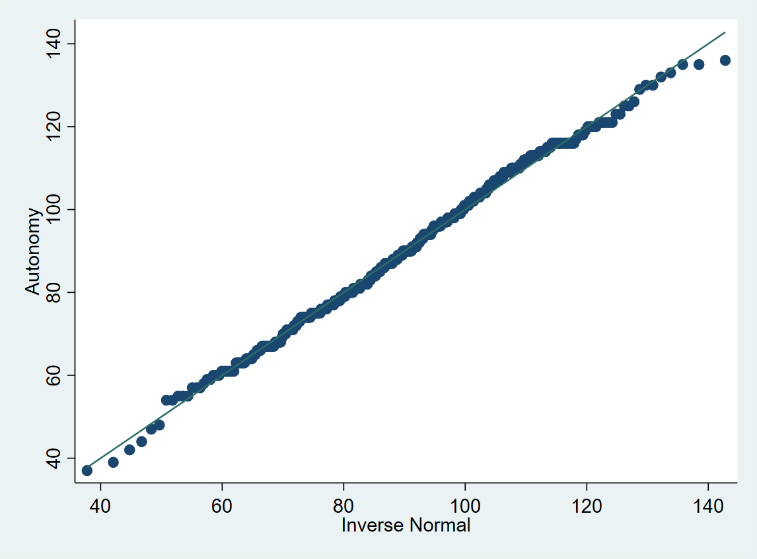

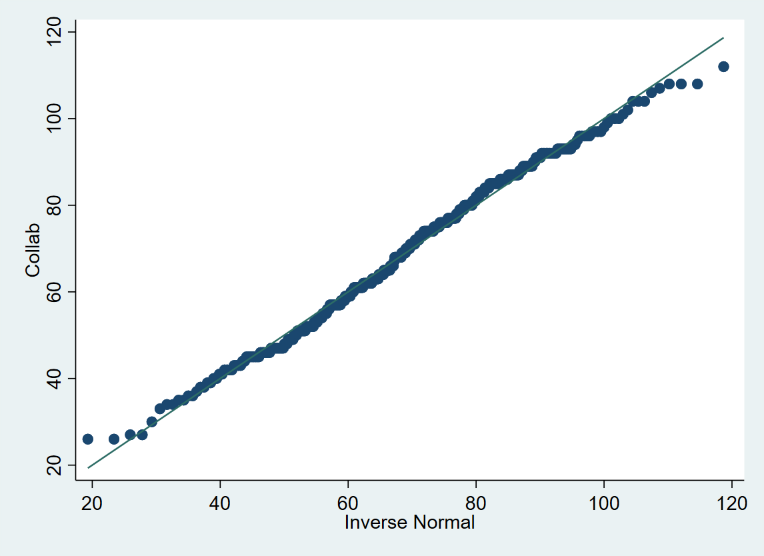
**Q-Q Plots**
2. **Collaboration 2. Autonomy**

**
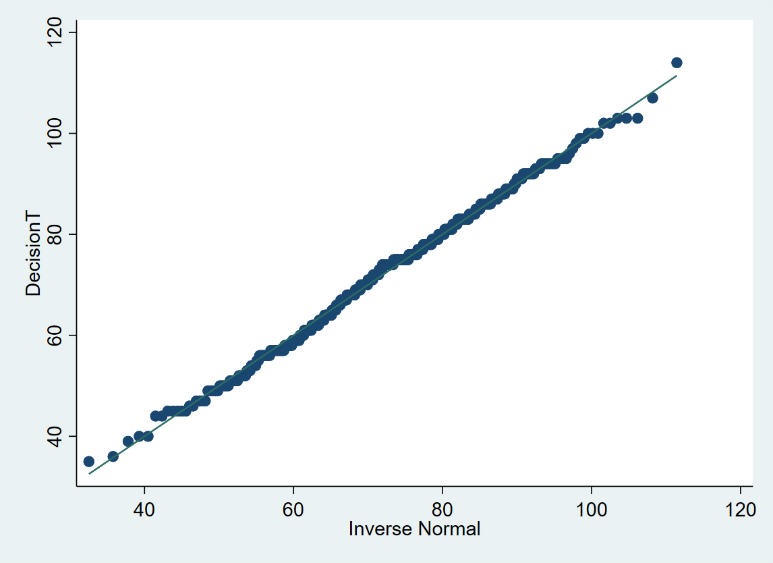
**

**3.** **Decision-making**

**C. Results of normality tests.**

| **Variable** | **Observation** | **W** | **V** | **z** | **Prob>z** |
| --- | --- | --- | --- | --- | --- |
| Collaboration | 400 | 0.991 | 2.396 | 2.079 | 0.019 |
| Decision-making | 400 | 0.997 | 0.935 | -0.159 | 0.563 |
| Autonomy | 400 | 0.995 | 1.295 | 0.616 | 0.269 |
