# Supplementary material for: Nurse–physician collaboration, autonomy, and decision-making ability among intensive care unit nurses in Tehran, Iran: A cross-sectional study
Source: PLoS One. 2025 Dec 9;20(12):e0336430. doi: 10.1371/journal.pone.0336430 (PMC12688092; doi:10.1371/journal.pone.0336430)
Supplement: Attachment — Submitted filename: Nurse-physician Collaboration Review Report.docx [file pone.0336430.s003.docx]

**Dear Editor and Authors,**

**I am grateful for the opportunity to review the paper "Nurse-physician Collaboration, Autonomy, and Decision-making Ability among Critical Care Nurses: A Cross-sectional Study." This study examines a highly relevant aspect of critical care nursing with a high sample size and standardized tools to determine significant correlations. Overall, the paper is well presented and clear to read. However, certain methodological, analytical, and interpretative issues must be resolved to enhance the validity of the findings and the overall impact of the paper.**

**- The abstract should contain a concise summary of the purpose and major findings. It should, nonetheless, mention the cross-sectional design in order to warn readers against drawing causal conclusions. Also, the fact that the final regression model explained only 14.2% of decision-making variance is a major finding that should be highlighted in the abstract to make the conclusion more balanced, indicating that there are other major determinants at play.**

**-The introduction does an excellent job of establishing the importance of collaboration, autonomy, and decision-making in the ICU environment. It is obvious that there is rationale for the study within the Iranian context. However, the relationship between these three constructs could be more theory-driven. While they are presented as interrelated, the hypothesized mechanism would be stronger if there were more detail on how they do this, i.e., how collaboration uniquely results in autonomy, or how they together enable improved decision-making.**

**-There are a number of strengths to the method section, including the use of validated tools and a large sample size. Of greater concern are the following concerns that need addressing:**

**-There is a significant discrepancy between planned sample size (n=180) and actual sample (n=400). The manuscript would be better if it included an explanation for this increased sample (e.g., increase in power for multivariate analysis, response rate that was better than anticipated) to ensure transparency.**

**-It states the goal was to have 400 participants and that 400 filled out the study, yet regression analysis is performed on 400 cases without mention of missing data treatment. If data were dropped, then how this was handled (e.g., listwise deletion) should be clearly stated, and any possible impact on selection bias should be noted in the limitations.**

**-While the Persian versions of the instruments are mentioned, a report of their psychometric properties in the Iranian or similar cultural group would be more convincing about the usage among this specific group.**

**-Results are reported clearly with elaborated tables. A particular finding must be addressed and interpreted more emphatically:**

**-The poor explained variance (R² = 14.2%) of the regression model is a most important result. Autonomy and collaboration are strong predictors, but findings indicate other factors account for over 85% of decision-making variance. This needs to be highlighted clearly and investigated in the main text of the results and then again in the discussion, rather than being presented as a simple statistic.**

**-The discussion effectively compares the levels of the main variables to other studies but could be enriched in several areas:**

**-The comment regarding a majority intuitive–analytic mode of decision making is intriguing. The discussion could be enhanced by relating this finding to established nursing theories, such as Benner's "Novice to Expert" model, to describe why this mixed mode is most suited to the high-tech ICU setting.**

**-The low R² must be explained in full. Acknowledge that autonomy and cooperation are good things but that presumably, there are a lot of other unmeasured variables (e.g., individual clinical skill, certain hospital protocols, leadership style, patient acuity) that must have a much greater impact on decision-making. This does not invalidate the results but puts them in a more realistic context.**

**-The effect of shift rotation on decision-making is dramatic. Theoretical accounts for this finding (e.g., variation in exposure to a wider variety of clinical presentations and team members) should be proposed.**

**-Limitations are accurately described as being the cross-sectional design and convenience sampling. The section should be lengthened to include:**

**-The single-site setting (two hospitals in Tehran), limiting the generalizability of the findings to other regions in Iran or to other countries with distinct health cultures.**

**-The reliance on self-reported data for all of the key constructs, subject to social desirability bias, especially within a hierarchical health culture.**

**-Direct mention of the low explained variance (R²) in the model as the key methodological flaw, indicating that the model is not adequate.**

**-The references list is adequate but can be complemented with newer studies from similar regional contexts for better positioning of the work. As an example, research reporting on resilience and stress in ICU nurses within the region (e.g., Aqtam et al. (2023) in INQUIRY) would strengthen background on the work environment in ICUs. Further, citations of clinical decision-making predictors literature from the Arab world (e.g., Aboalrob et al. (2025) in PLOS ONE) would be excellent comparison knowledge and indicate a broader utilization of local literature.**

**Aqtam, I., Ayed, A., Toqan, D., et al. (2023). The relationship between stress and resilience of nurses in intensive care units during the COVID-19 pandemic. INQUIRY: The Journal of Health Care Organization, Provision, and Financing, 60.** <https://doi.org/10.1177/00469580231179876>

**Aboalrob, W., Ayed, A., Malak, M. Z., & Aqtam, I. (2025). Understanding the influence of self-concept on clinical decision-making among nurses: A cross-sectional study. PLOS ONE, 20(8), e0330905.** <https://doi.org/10.1371/journal.pone.0330905>

**This is a valuable piece of work with the potential to make a significant contribution to critical care nursing practice. The key findings regarding the significance of autonomy and collaboration are compelling. By responding to issues outlined above, particularly clarifying rationale for sample size, offering more interpretation of the low R², widening the theoretical and regional scope, and firmly acknowledging limitations, the authors can produce a much improved manuscript. I believe these revisions are required for the piece to be at its finest.**

**I recommend Major Revision.**
