# Supplementary material for: Nurse–physician collaboration, autonomy, and decision-making ability among intensive care unit nurses in Tehran, Iran: A cross-sectional study
Source: PLoS One. 2025 Dec 9;20(12):e0336430. doi: 10.1371/journal.pone.0336430 (PMC12688092; doi:10.1371/journal.pone.0336430)
Supplement: Attachment — Submitted filename: Re (3).docx [file pone.0336430.s005.docx]

| **No.** | **Comment** | **Response and explanation** | **Location in Manuscript** |
| --- | --- | --- | --- |
| **Editor** | | | |
| We sincerely thank the editor for their careful handling of our manuscript and for providing insightful guidance throughout the review process. We greatly appreciate the time and effort invested in coordinating the peer review and facilitating constructive feedback, which has substantially contributed to improving the clarity, rigor, and overall quality of our manuscript. | | | |
| **Reviewer 1** | | | |
| Introduction | Incorporating recent regional studies will enhance relevance and underscore the multidimensionality of decision-making. This insertion would fit at the end of the Introduction section, just before stating the study aim. | We appreciate the reviewer’s valuable suggestion. In response, we have incorporated recent regional studies to enhance the contextual relevance and highlight the multidimensional nature of decision-making. | 3-7 |
|  | Recent evidence highlights the importance of individual psychosocial factors in shaping clinical decision-making among nurses. For example, Aboalrob et al. (2025) demonstrated that nurses’ self-concept significantly influences their ability to make sound clinical decisions, underscoring the need to consider personal and professional identity in decision-making research. Similarly, Ayed (2025) investigated the relationship between emotional intelligence and decision-making among NICU nurses, reporting that higher emotional intelligence was positively associated with better decision-making competence. These findings suggest that beyond organizational structures, individual-level attributes such as self-concept and emotional intelligence interact with professional autonomy and interprofessional collaboration to shape decision-making ability. | We appreciate the reviewer’s insightful comment. Following this suggestion, we have incorporated recent regional evidence to enhance the relevance and emphasize the multidimensional nature of nurses’ clinical decision-making. Specifically, we added a paragraph highlighting recent findings from Aboalrob et al. (2025) and Ayed (2025), which discuss the role of self-concept and emotional intelligence in shaping nurses’ decision-making competence. | 3-7 |
| Methods | The convenience sampling from two hospitals may limit external validity. Please clarify how many ICUs/units were covered in each hospital and whether participants were evenly distributed. | We thank the reviewer for this important point. To clarify, participants were recruited from the critical-care units of two tertiary university hospitals affiliated with Shahid Beheshti University of Medical Sciences (Imam Hossein and Loghman Hakim). We have added a short paragraph to the Methods (Study Design and Setting) that specifies the number of ICU units sampled at each hospital and the exact participant distribution by hospital and unit. | 7 |
|  | Instruments  More detail is needed on the cultural adaptation process of the Lauri & Salanterä tool in Persian ICU contexts. | Thank for the reviewer’s valuable and constructive comment. We appreciate the time and expertise invested in improving our manuscript; the suggestion helped us clarify the manuscript’s regional relevance and strengthen the Methods and Instruments sections. We have incorporated the recommended changes and believe they have meaningfully improved the paper. | 12 |
| Results and Discussions | Please emphasize why autonomy scores were higher in this sample compared to previous studies, and discuss how local ICU culture, nurse–physician hierarchy, or hospital type may have contributed. Avoid overemphasis on non-significant demographic findings. | We thank the reviewer for this important comment. We agree that the higher autonomy scores observed in our sample require additional contextual explanation. In response, we have expanded the Discussion to clarify plausible reasons for this finding and to situate it within the regional and ICU-specific literature. Specifically, we added a paragraph that (a) highlights how ICU practice characteristics and local ICU culture can foster greater autonomy, (b) discusses the potential influence of nurse–physician hierarchies and hospital type (e.g., teaching versus non-teaching) on perceived autonomy, and (c) explicitly acknowledges that non-significant demographic associations (e.g., age, experience) were not emphasized in our interpretation. | 28-29 |
| Implications | Implications for practice and policy should be more practical and context-specific. For instance, could structure interprofessional rounds, shared governance, or simulation-based training realistically be implemented in Tehran ICUs? Educational recommendations should emphasize decision-making training that integrates intuitive and analytical reasoning, as noted in recent regional literature. | Thank you for this helpful suggestion. We have revised the Implications section to include practical, context-specific recommendations for Tehran ICUs, detailing feasible organizational steps (pilot shared governance, time-protected interprofessional case-review forums, nurse and physician champions), educational strategies that integrate intuitive and analytical reasoning (simulation, reflective rounds, mentorship), and concrete performance indicators for monitoring. | 32-34 |
| Limitation | The limitations section should be expanded to include Self-report bias | We thank the reviewer for highlighting this important methodological limitation. In response, we have expanded the Limitations subsection to explicitly acknowledge the potential for self-report bias and its implications. | 30-31 |
| **Reviewer 2** | | | |
| **I am grateful for the opportunity to review the paper "Nurse-physician Collaboration, Autonomy, and Decision-making Ability among Critical Care Nurses: A Cross-sectional Study." This study examines a highly relevant aspect of critical care nursing with a high sample size and standardized tools to determine significant correlations. Overall, the paper is well presented and clear to read. However, certain methodological, analytical, and interpretative issues must be resolved to enhance the validity of the findings and the overall impact of the paper.**  **Re:**  We wish to express our sincere and profound gratitude for the time, care, and scholarly insight you invested in reviewing our manuscript entitled “Nurse-physician Collaboration, Autonomy, and Decision-making Ability among Critical Care Nurses: A Cross-sectional Study.” Your thoughtful and constructive comments have materially strengthened the clarity, rigor, and contextualization of our work. We have carefully considered each point you raised and made corresponding revisions to the manuscript. Below, we provide a detailed, point-by-point response and indicate where changes have been made in the revised version. We are grateful for your guidance and remain available to address any further suggestions. | | | |
| 1. | The abstract should contain a concise summary of the purpose and major findings. It should, nonetheless, mention the cross-sectional design in order to warn readers against drawing causal conclusions.  Also, the fact that the final regression model explained only 14.2% of decision-making variance is a major finding that should be highlighted in the abstract to make the conclusion more balanced, indicating that there are other major determinants at play. | We thank the reviewer for this helpful observation. The Abstract has been revised to provide a more concise summary of the study’s purpose and main findings, while explicitly stating that the research employed a cross-sectional design. This clarification aims to prevent readers from inferring causality from the observed associations.  We appreciate the reviewer’s insightful comment. In response, the Abstract has been revised to explicitly highlight that the final regression model explained only 14.2% of the variance in clinical decision-making. This addition helps present a more balanced interpretation, acknowledging that while autonomy and collaboration were significant predictors, other unmeasured factors also contribute to decision-making among ICU nurses. | 2 |
| 2. | The introduction does an excellent job of establishing the importance of collaboration, autonomy, and decision-making in the ICU environment. It is obvious that there is rationale for the study within the Iranian context. However, the relationship between these three constructs could be more theory-driven. While they are presented as interrelated, the hypothesized mechanism would be stronger if there were more detail on how they do this, i.e., how collaboration uniquely results in autonomy, or how they together enable improved decision-making. | Thank you for the constructive comment. We have strengthened the theoretical framework in the Introduction and clarified the proposed mechanism linking collaboration, autonomy, and decision-making based on evidence published since 2020 (including studies on Relational Coordination, psychological empowerment, and naturalistic decision-making models). We have also specified mediation and moderation hypotheses for empirical testing. | 3-7 |
| 3. | There are a number of strengths to the method section, including the use of validated tools and a large sample size. Of greater concern are the following concerns that need addressing:  -There is a significant discrepancy between planned sample size (n=180) and actual sample (n=400). The manuscript would be better if it included an explanation for this increased sample (e.g., increase in power for multivariate analysis, response rate that was better than anticipated) to ensure transparency. | Sample size of 180 was calculated a priori based on an anticipated correlation of 0.242 (α=0.05, power=90%). During the enrolled recruitment period (18 August–8 September 2025), we obtained 400 eligible and high-quality responses. This increase arose from an unexpectedly high participation rate following active endorsement by head nurses, the use of widely accessible online platforms (WhatsApp and Bale) for questionnaire distribution, and survey settings that required complete responses and prevented duplicate submissions. We note that the larger sample increases the precision of estimated effects and the statistical power for multivariable and mediation models, and enables planned subgroup analyses. We have added a brief explanatory paragraph to the Methods to improve transparency | 9 |
| 4 | -It states the goal was to have 400 participants and that 400 filled out the study, yet regression analysis is performed on 400 cases without mention of missing data treatment. If data were dropped, then how this was handled (e.g., listwise deletion) should be clearly stated, and any possible impact on selection bias should be noted in the limitations. | Thank you for this critical observation. For clarity, the online survey was configured with mandatory item completion, and following quality-control screening for duplicate or low-quality responses, no missing data remained in the analytic dataset. Therefore, all 400 valid responses were included in the regression analyses, and no cases were removed nor was imputation required. Nevertheless, we have acknowledged the potential for selection/response bias related to convenience sampling and mandatory completion in the Limitations section. | 9 |
| 5 | -While the Persian versions of the instruments are mentioned, a report of their psychometric properties in the Iranian or similar cultural group would be more convincing about the usage among this specific group. | Thank you for the reviewer’s valuable and constructive comment. We appreciate the time and expertise invested in improving our manuscript; the suggestion helped us clarify the manuscript’s regional relevance and strengthen the Methods and Instruments sections. We have incorporated the recommended changes and believe they have meaningfully improved the paper. | 10-12 |
| 6 | Results are reported clearly with elaborated tables. A particular finding must be addressed and interpreted more emphatically:  -The poor explained variance (R² = 14.2%) of the regression model is a most important result. Autonomy and collaboration are strong predictors, but findings indicate other factors account for over 85% of decision-making variance. This needs to be highlighted clearly and investigated in the main text of the results and then again in the discussion, rather than being presented as a simple statistic. | Thank you for highlighting the importance of the low R². We have revised the Results to emphasize that the whole model explains ~14% of the variance and added a focused Discussion section to interpret this finding. We now explicitly note that, although autonomy and collaboration were significant predictors, most variance in decision-making remains unexplained and is likely attributable to additional individual (e.g., emotional intelligence, fatigue), situational (patient acuity, time pressure), and organizational (staffing, leadership, protocols) factors. We have added recommendations for future research (longitudinal, mixed-methods, objective performance measures, SEM or multilevel modelling) and acknowledged this issue in the Limitations. | 15-23 |
| 7 | Limitations are accurately described as being the cross-sectional design and convenience sampling. The section should be lengthened to include:  -The single-site setting (two hospitals in Tehran), limiting the generalizability of the findings to other regions in Iran or to other countries with distinct health cultures.  -The reliance on self-reported data for all of the key constructs, subject to social desirability bias, especially within a hierarchical health culture.  -Direct mention of the low explained variance (R²) in the model as the key methodological flaw, indicating that the model is not adequate. | We sincerely thank the reviewer for this insightful comment and for suggesting ways to strengthen the transparency and completeness of the Limitations section. In response, we have revised and expanded this section to explicitly address the additional methodological constraints noted by the reviewer. | 30-31 |
|  | The discussion effectively compares the levels of the main variables to other studies but could be enriched in several areas:  -The comment regarding a majority intuitive–analytic mode of decision making is intriguing. The discussion could be enhanced by relating this finding to established nursing theories, such as Benner's "Novice to Expert" model, to describe why this mixed mode is most suited to the high-tech ICU setting.  -The low R² must be explained in full. Acknowledge that autonomy and cooperation are good things but that presumably, there are a lot of other unmeasured variables (e.g., individual clinical skill, certain hospital protocols, leadership style, patient acuity) that must have a much greater impact on decision-making. This does not invalidate the results but puts them in a more realistic context.  -The effect of shift rotation on decision-making is dramatic. Theoretical accounts for this finding (e.g., variation in exposure to a wider variety of clinical presentations and team members) should be proposed. | We are grateful to the reviewer for their constructive suggestions on enriching the Discussion. In response, we have expanded the Discussion to (1) relate the predominance of an intuitive–analytic decision-making mode to established nursing theory (Benner’s Novice-to-Expert framework), (2) provide a fuller explanation for the low explained variance (R² = 0.142) by acknowledging likely unmeasured determinants, and (3) offer theoretical accounts for the observed association between rotating shifts and higher decision-making scores. |  |
| 9 | -The references list is adequate but can be complemented with newer studies from similar regional contexts for better positioning of the work. As an example, research reporting on resilience and stress in ICU nurses within the region (e.g., Aqtam et al. (2023) in INQUIRY) would strengthen background on the work environment in ICUs. Further, citations of clinical decision-making predictors literature from the Arab world (e.g., Aboalrob et al. (2025) in PLOS ONE) would be excellent comparison knowledge and indicate a broader utilization of local literature.  Aqtam, I., Ayed, A., Toqan, D., et al. (2023). The relationship between stress and resilience of nurses in intensive care units during the COVID-19 pandemic. INQUIRY: The Journal of Health Care Organization, Provision, and Financing, 60. https://doi.org/10.1177/00469580231179876 | We appreciate the reviewer’s valuable recommendation to strengthen the regional contextualization of the manuscript. In response, we have cited the suggested studies and incorporated relevant regional literature to enhance the background and discussion. | Whole |
| **Reviewer 3** | | | |
| Title | 1. The title is clear and reflects the scope, but the phrase “A Cross-sectional Study” could be expanded with a more specific methodological explanation or mention of the country (e.g., “in Iran,” etc.). | We thank the reviewer for this helpful suggestion regarding the title. In response, we have revised the title to make it more specific and clearly indicate the study context. | 1 |
| Introduction | 1. The introduction presents the topic of “decision-making in critical care nurses” with very broad transitions; key concepts (e.g., “intuitive–analytic decision-making”) should be defined first, followed by their place in the literature. | We sincerely thank the reviewer for this insightful comment. We agree that a more evident conceptual progression and explicit definitions of key constructs would strengthen the Introduction. In response, we have revised the early part of the Introduction to define clinical decision-making and intuitive–analytic decision-making more precisely, and to clarify their relevance within critical care nursing, before transitioning to the discussion of autonomy and collaboration. | 3-7 |
|  | 2. Although the literature review is comprehensive, some sections are written using too many first-person pronouns or vague expressions; they should be presented using more objective, straightforward sentences. | We appreciate the reviewer’s valuable observation regarding the use of first-person pronouns and vague expressions in certain parts of the literature review. We fully agree that an objective and formal tone enhances academic clarity and aligns with the conventions of scientific writing.  In response, we have carefully reviewed the entire Introduction and Background/Literature Review sections. We have removed first-person expressions (e.g., “we aimed,” “our study focuses,” “we believe”) and replaced them with more objective, impersonal phrasing. Additionally, we have refined several sentences to improve precision and eliminate vague wording. | 3-7 |
|  | 3. The conceptual framework should be clarified with a model or diagram; a logical flow should be added. The connection between decision-making, autonomy, and collaboration should be shown more clearly. | We sincerely thank the reviewer for this insightful and constructive suggestion. We agree that presenting a clear conceptual framework strengthens the logical flow and helps readers better understand the theoretical connection among nurse–physician collaboration, professional autonomy, and clinical decision-making ability.  In response, we have developed and inserted a conceptual model diagram in the Introduction section (final paragraph) to visually represent the hypothesized relationships among the study variables. The accompanying paragraph explicitly describes the model’s logic and theoretical rationale. | 3-7 |
|  | 4. In the literature review, a brief definition should be provided for each key concept (collaboration, autonomy, decision-making), and emphasis should be placed on why these are important for ICU nurses. | We thank the reviewer for this valuable suggestion. To address it, we have revised the literature review to include brief definitions for each key concept and emphasized their relevance for ICU nurses: | 3-7 |
|  | 5. The research gap and problem definition presented are not expressed in a sufficiently direct and original manner; for example, comparisons with the Iranian context or previous studies are made arbitrarily, and specific reasons and shortcomings should have been emphasized more sharply. | We thank the reviewer for highlighting the need to express the research gap and problem definition more clearly. To address this concern, we have revised the introduction to explicitly emphasize the rationale for our study in the Iranian ICU context. Specifically, we have added the following statement to the final paragraph of the introduction:  "Although previous studies have explored nurse autonomy, inter-professional collaboration, and decision-making abilities, few have examined their interaction specifically within the Iranian ICU context. Considering the unique organizational hierarchies, cultural factors, and resource constraints in Iranian hospitals, understanding these relationships is essential for designing targeted interventions to empower nurses and improve patient care outcomes."  This addition directly addresses the research gap, highlights the specific context of Iran, and underscores the necessity of our study. | 3-7 |
|  | 6. A specific paragraph stating “the purpose of the study” should be added directly at the end of the introduction; this is not clearly distinguished in the current text. The sentence “Therefore, this study was conducted” should be directly linked to the purpose, and if high-impact results are expected, these should be clearly addressed in the text. | We thank the reviewer for this helpful comment. To address it, we have added a specific paragraph at the end of the introduction explicitly stating the purpose of the study and linking it to expected high-impact results. | 3-7 |
|  | 7. The research question or hypotheses section should be formulated clearly and concisely at the end of the introduction. | We thank the reviewer for this important suggestion. To address it, we have added a concise section at the end of the introduction, clearly stating the research questions and hypotheses. | 3-7 |
|  | 8. The reference list is insufficient, especially in terms of new and local studies; it should be further supported by current literature (last 2 years) and should be based on local data as well as classical studies. | We thank the reviewer for this important suggestion. To address it, we have updated the reference list by:   1. Including recent studies from the last two years to ensure the literature reflects current evidence. 2. Adding local studies from Iran to contextualize the findings and strengthen the relevance of the research in the Iranian ICU setting. 3. Retaining classical and foundational studies to support key theoretical frameworks and concepts. |  |
|  | 9. Comparisons with international literature are particularly lacking in the citation system; for example, findings from other countries should be added in addition to “previous Iranian studies.” | We thank the reviewer for this valuable comment. To address it, we have revised the manuscript to include comparisons with international literature in addition to previous Iranian studies. | 3-7 |
| Method | 1. The study design is described as “cross-sectional analytical,” but despite being multicenter, it does not explicitly discuss the limitation of generalizability. | We thank the reviewer for this important comment. Although our study was conducted in two major teaching hospitals in Tehran and included eight ICUs, we acknowledge that the findings may not be fully generalizable to all ICU settings in Iran or other countries due to differences in organizational structures, patient populations, and clinical practices. This limitation has now been explicitly stated in the Discussion section. | 30-31 |
|  | 2. Participant selection was performed using convenience sampling; this issue has not been sufficiently addressed in terms of bias risk, external validity limitations, and sample representativeness. The exclusion criteria (“straight-lining,” “duplicate registration”) lack methodological justification; the extent to which they ensure sample integrity is debatable. Sample selection and exclusion criteria should be discussed in more detail in terms of cause-and-effect relationships. | We thank the reviewer for this insightful comment. We acknowledge that using convenience sampling may introduce selection bias and limit the external validity of our findings, as participants may not fully represent all ICU nurses in Iran. This limitation has now been explicitly addressed in the Discussion section.  Regarding the exclusion criteria, measures such as removing responses with “straight-lining” (indicating inattentive or random answering) or “duplicate registration” were implemented to ensure data quality and sample integrity. These criteria are standard in survey-based research to minimize measurement errors and improve the reliability of results. We have clarified in the manuscript that these procedures were not intended to exclude participants arbitrarily, but rather to maintain the methodological rigor and validity of the collected data.  Finally, we have added a discussion on how the sampling method and exclusion criteria may influence the interpretation of cause-and-effect relationships, emphasizing that due to the cross-sectional design, causal inferences are limited. | 30-31 |
|  | 3. Only online completion was used in the data collection process, and the possible impact of this method on social desirability and response bias was not mentioned. | We thank the reviewer for highlighting this critical point. We acknowledge that using exclusively online questionnaires may introduce response biases, including social desirability bias, as participants might provide answers they perceive as favorable. To mitigate this, participants were assured of anonymity and confidentiality, and clear instructions were provided to encourage honest and thoughtful responses. This limitation has now been explicitly acknowledged in the limitation section. | 25-26 |
|  | 4. The data collection procedure (“mandatory online completion”) carries risks of accuracy and bias due to the sample; the possibility of social desirability bias should be discussed. The fact that participants submitted only once from the same IP address does not in itself ensure reliability. Strategies for addressing the risks of bias in online data collection should be clearly stated. | We thank the reviewer for this valuable comment. We acknowledge that mandatory online completion and limiting submissions to a single IP address could introduce response biases and do not, by themselves, fully ensure data reliability. To address these risks, several strategies were implemented: participants were assured of anonymity and confidentiality, instructions emphasized honest and thoughtful responses. Additionally, exclusion criteria such as straight-lining and duplicate registrations were applied to improve data quality. These measures collectively aimed to minimize social desirability bias and enhance the reliability of the online data collection. | 30-31 |
|  | 5. The validations of the scales used are well explained, but validity-reliability issues that may arise when applied in different cultures and local samples have not been fully addressed. | We thank the reviewer for highlighting this important issue. While the Persian versions of the P-AITCS-II, DPBS, and LSCD-MQ scales have demonstrated acceptable validity and reliability in previous Iranian studies, we acknowledge that applying these instruments in different cultural and organizational contexts may still introduce limitations. We have now explicitly discussed this point in the Methods section, noting that cultural factors, language nuances, and local ICU practices could influence responses. | 30-31 |
|  | 6. The demographic survey is broad in scope, but the role of the variables obtained in the analysis plan is not sufficiently explained. | We thank the reviewer for this comment. While our demographic questionnaire collected comprehensive information, only those demographic variables that demonstrated statistically significant associations in preliminary analyses (p < 0.05) were included as covariates in the multivariable analyses. This approach ensured that the analyses accounted for potential confounding effects while focusing on variables meaningfully related to nurse-physician collaboration. This clarification has been added to the Methods section. | 14 |
|  | 7. It is positive that a power analysis was performed, but the sample size does not contribute to generalizability in practice, as it is limited to two hospitals. | We thank the reviewer for this comment. We acknowledge that although a power analysis was performed and the sample size was sufficient for statistical analyses, the study was conducted in only two hospitals. Therefore, the findings may have limited generalizability to other ICU settings. This limitation has been explicitly discussed in the Limitations section of the manuscript. | 30-31 |
|  | 8. Normal distribution checks and verification methods are explained, but alternative tests to be applied in case of deviation are not specified. | We thank the reviewer for this comment. Normality checks were performed, and the results are presented in Supplementary Appendix 2. Based on these analyses, all data were considered to meet the assumptions of normality. Therefore, parametric tests were appropriately applied. In cases where deviations from normality might occur, we acknowledge that non-parametric alternatives could be considered, although they were not required in this dataset. | 14 |
|  | 9. In multiple regression, the variables included in the model are clearly listed. Still, the rationale for not including additional variables is lacking, as the model's variance explanation rate is low (approximately 14%). | We thank the reviewer for this comment. The rationale for including specific variables in the multiple regression model is provided in the Methods section, where it is explained that only demographic variables showing statistically significant associations in preliminary analyses (p < 0.05) were included. We acknowledge that the model’s variance explanation rate is approximately 14%, which is consistent with the complexity of factors influencing decision-making. | 14 |
|  | 10. The SPSS version is specified, but the analysis processes are not detailed; for example, details such as which variables were used in which analysis and which control variables were applied for correlation are unclear. The statistical analysis section should provide detailed subheadings for all methods and control variables used. Additional control steps regarding the samples should be written to ensure the adequacy of the data set and its resistance to manipulation. | We thank the reviewer for this important suggestion. The Statistical Analysis section has been revised to include detailed subheadings specifying all analyses performed, including descriptive statistics, normality checks, correlation analyses, and multiple regression models. The variables included in each analysis, along with the control variables (demographic covariates with significant associations in preliminary analyses), are now clearly stated. | 14 |
|  | 11. The ethical approval and consent process is clearly explained, but the steps taken for data anonymization and confidentiality are not described in technical detail. | We thank the reviewer for this comment. While the ethical approval and informed consent process were clearly described, we have now provided additional technical details regarding data anonymization and confidentiality. All responses were collected via an online platform that did not record personally identifiable information. Each participant was assigned a unique study ID, and the dataset was stored on a secure, password-protected server accessible only to the research team. These measures ensured that participants’ identities remained confidential and that data were handled in accordance with ethical standards. | 13 |
|  | 12. Restrictions on data sharing are specified, but there is no additional information regarding access to raw data and the verifiability of the analyses. | We thank the reviewer for this comment. While data sharing restrictions were noted, we clarify that the raw dataset supporting the findings of this study is securely stored and can be made available upon reasonable request to the corresponding author, subject to ethical approvals and institutional regulations. Additionally, the analysis scripts and procedures used in SPSS are fully documented, ensuring that the results are verifiable and reproducible by qualified researchers. | 35 |
| Results | 1. The findings were initially presented using descriptive statistics (socio-demographic characteristics, means, standard deviations), but in their current form, they provide excessive detail; the main text includes unnecessary repetition in the explanations of the tables. | We appreciate the reviewer’s observation regarding the level of detail in the Results section. We have carefully revised the text to eliminate redundancy and excessive repetition of table contents. Specifically, the descriptions of socio-demographic characteristics and primary study variables have been condensed, focusing on the key findings. Detailed numerical data are retained in the tables for reference, while the main text now emphasizes summary interpretations to enhance clarity and readability. | 15-23 |
|  | 2. Under each subheading (e.g., correlation analysis, group comparisons, regression), the coherence between text and tables is weak; the matching of findings to tables and the explanatory notes should be improved. | We thank the reviewer for highlighting the need for improved coherence between the text and tables. We have carefully revised the Results section to ensure that each subheading (correlations, group comparisons, regression) directly reflects the corresponding table. Key findings are now clearly summarized in the text, and detailed numerical data remain in the tables for reference. We also added explanatory notes where necessary to clarify the relationship between the findings and their statistical representation. | 15-23 |
|  | 3. The flow of findings is disjointed; it is not clearly emphasized which analysis responds to which hypothesis. | We appreciate the reviewer’s comment regarding the flow of the Results section. We have revised the section to explicitly link each analysis to the corresponding hypothesis. Subheadings have been reorganized, and transitions have been added to clarify which findings address which hypotheses. This improves the logical flow and makes it easier for readers to follow the study’s analytic framework. | 15-23 |
|  | 4. Table titles and variable descriptions are sometimes missing or confusing; in particular, sub-dimension names and abbreviations should be clear to an international audience. | We thank the reviewer for highlighting the need for clearer table titles and variable descriptions. We have revised all tables to ensure that each title accurately reflects the content. Sub-dimension names and abbreviations are now fully spelled out and explained in either the table footnotes or the main text, making them clear and understandable to an international audience. | 15-23 |
|  | 5. References to tables and figures are not provided appropriately between findings; clear references to tables should be made within the text (e.g., “See Table 2”). | We thank the reviewer for pointing out the need for explicit references to tables and figures. We have revised the Results section to ensure that every key finding is directly linked to the relevant table or figure. References, such as “see Table 2” or “as shown in Figure 1,” have been consistently added to improve readability and enable readers to locate the detailed data easily. | 15-23 |
|  | 6. Descriptive statistics only provide the mean and SD; in some cases, percentages and frequency distributions should also be included. | We appreciate the reviewer’s comment regarding the inclusion of percentages and frequency distributions. The instruments used in this study do not have established cut-off points for categorizing scores; therefore, descriptive statistics for the primary constructs are appropriately presented using means and standard deviations. However, frequency distributions and percentages have been provided for all sociodemographic and work-related variables (see Table 1) to give readers a clear overview of the sample characteristics. Also, for decision-making, we categorized the data and reanalyzed as per your recommendation. | 15-32 |
|  | 7. The explanatory power of the multiple regression analysis is low (14.2%), but the text lacks a reference to the reason for this or to the limitations section. | We appreciate the reviewer’s observation regarding the explanatory power of the regression model. We have added a clarification in the Results section noting that the model explained 14.2% of the variance in Decision-Making and referred readers to the Limitations section, where we discuss potential reasons for this moderate R² value, such as the complexity of psychosocial and behavioral factors influencing decision-making that the included predictors do not fully capture. | 25-26 |
|  | 8. The practical meaning of both descriptive and inferential statistics should be contextualized with short summary sentences; for example, the relationship between statistically significant differences and clinical/practical meaning should have been discussed. | We thank the reviewer for this important observation. We have revised the Results section to include brief contextual interpretations of both descriptive and inferential statistics. Key findings, such as the relationship between shift type and Decision-Making, are now accompanied by short sentences highlighting their practical or clinical significance, in addition to reporting statistical significance. | 15-23 |
|  | 9. The tables and statistical analyses are adequate, but in some places the descriptive statistics take up too much space, and the findings section is unnecessarily long. The findings should be condensed. | We thank the reviewer for this suggestion. We have condensed the Findings section by summarizing descriptive statistics and highlighting key results, while retaining detailed numerical data in tables for reference. Repetitive information has been removed, making the text more concise and readable without losing essential content. | 15-23 |
|  | 10. The regression model explains only 14.2% of the variance; this indicates that other important variables (organizational culture, managerial support, workload, etc.) are missing and should be clearly discussed. | We thank the reviewer for highlighting the relatively low explanatory power of the regression model. We have added a discussion in the Limitations section noting that the model explains only 14.2% of the variance in Decision-Making. We explicitly acknowledge that other important factors, such as organizational culture, managerial support, workload, and additional psychosocial variables, were not included in this study and may contribute to Decision-Making. Future research should consider these factors to develop a more comprehensive model. | 30-31 |
|  | 11. Some variable names in the table presentations are mistranslated or confusing (e.g., “Collaboration” and its sub-dimensions). Internationally accepted titles and abbreviation definitions should be added for tables and graphs. | We thank the reviewer for this valuable comment. We have carefully reviewed all table and figure titles, as well as variable names, to ensure that they are accurately translated and clearly understandable for an international audience. Sub-dimensions and abbreviations of constructs such as Collaboration, Autonomy, and Decision-Making have been fully spelled out and described in the table footnotes or figure legends. | Tables |
|  | 12. Some tables and appendices are not fully integrated into the main text. | We thank the reviewer for this observation. We have revised the manuscript to ensure that all tables and appendices are appropriately integrated and referenced in the main text. Each table and appendix is now cited at the appropriate point in the Results or Methods sections, making it clear to the reader where the detailed information can be found. | Tables |
|  | 13. Some findings (e.g., the relationship between educational status and decision-making) are said to be inconsistent with previous studies, but the reasons are not sufficiently discussed. | We thank the reviewer for pointing out the inconsistency between our findings and previous studies regarding the role of educational level in clinical decision-making. We have added a clarification in the Discussion section explaining that, in the Iranian context, the number of nurses with advanced degrees (Master’s or PhD) working in clinical settings is relatively small, as most of these individuals are employed in academic or teaching positions rather than at the bedside. This contextual factor likely limits the observable influence of formal education on clinical decision-making in our sample. | 23-29 |
|  | 14. The results regarding differences between different groups (e.g., shift type, educational status) are left without justification, i.e., the literature review related to the findings is lacking. | We sincerely thank the reviewer for this valuable comment. We have revised the manuscript by adding relevant references and discussion for all group differences reported in the Results section (e.g., shift type, educational status), providing justification based on previous literature as requested. | 23-29 |
|  | 15. The finding of no relationship between demographic variables and main variables is presented without discussion; however, the homogeneity of the sample or any sampling bias is not addressed. | We sincerely thank the reviewer for this comment. We have added a discussion clarifying that the lack of significant relationships between demographic variables and the main constructs may be attributed to the relative homogeneity of our sample and potential sampling characteristics. This explanation has been incorporated into the revised manuscript to address the reviewer’s concern. | 19 |
|  | 16. If there are findings/statistics regarding the limitations of “self-report” data, they are not emphasized; possible biases such as social desirability bias could be briefly mentioned here before leaving them for discussion. | We sincerely thank the reviewer for this comment. We have emphasized in the Limitations section that the use of self-report data may introduce biases, including social desirability bias, and this point has now been clearly highlighted in the revised manuscript. | 30-31 |
|  | 17. Findings/analysis processes should be titled according to the hypothesis or research questions (e.g., “H1: Relationship between ... and ...”, “H2: Difference between ... groups”, etc.). | We sincerely thank the reviewer for this suggestion. Subheadings in the Results section have now been revised and aligned with the corresponding hypotheses and research questions as requested. | 15-23 |
|  | 18. In addition to significance values (p), additional statistical indicators such as effect size (Cohen’s d, OR, Beta) and confidence intervals should be provided if available. | We sincerely thank the reviewer for this comment. Where applicable, additional statistical indicators, including effect sizes (e.g., Cohen’s d, standardized Beta) and confidence intervals, have been added to the Results section to provide a more comprehensive interpretation of the findings. | 15-23 |
| Discussion | 1. The main findings are repeated but not discussed in a focused manner according to the research question or hypotheses; that is, a systematic analysis of the findings according to the predetermined objectives/hypotheses is lacking. | We sincerely thank the reviewer for this insightful comment. We have revised the Results section to systematically present and discuss the findings in accordance with the research questions and hypotheses, providing a focused and structured analysis as requested. | 23-29 |
|  | 2. Although literature comparisons were adequately performed, the country-specific (Iran) system, cultural, and institutional differences that could lead to similar/different findings should have been discussed more robustly. | We sincerely thank the reviewer for this valuable comment. We have strengthened the Discussion section by highlighting country-specific (Iranian) systemic, cultural, and institutional factors that may account for similarities or differences in our findings compared to previous studies. | 23-29 |
|  | 3. Contradictory results in the literature are approached only at the level of “we agree/disagree”; the possible contradictions between the findings and contextual reasons are not explored in depth. | We sincerely thank the reviewer for this insightful comment. We have revised the Discussion section to explore possible contradictions between our findings and previous literature more thoroughly, including consideration of contextual and situational factors that may explain these discrepancies. | 23-29 |
|  | 4. The limitations section is presented positively; however, methodological constraints such as the “self-report” method, sample representativeness, and social desirability bias should be supported with more concrete data and examples. | We sincerely thank the reviewer for this valuable comment. We have revised the Limitations section to more clearly address methodological constraints, including the use of self-report data, sample representativeness, and potential social desirability bias, providing additional clarification as appropriate. | 30-31 |
|  | 5. The ability to generalize the findings should be discussed in detail; the sample structure and distribution should be elaborated beyond the statement that it is “limited to two hospitals.” | We sincerely thank the reviewer for this comment. We have revised the Limitations section to provide a more detailed discussion of the generalizability of the findings, including elaboration on the sample structure and distribution beyond the fact that data were collected from two hospitals. | 30-31 |
|  | 6. The recommendations provided for future research are too general and do not directly address the findings and limitations of the study; more specific, actionable, and gap-filling recommendations are needed (e.g., incorporating organizational factors into future models, supporting them with observational data).  7. Repetition with large-scale, multi-center, and/or multi-regional samples should be recommended. | We sincerely thank the reviewer for this important suggestion. We have revised the Recommendations for Future Research to provide more specific and actionable guidance directly linked to the study findings and limitations. In particular, we now suggest incorporating organizational factors into predictive models, complementing self-report instruments with objective observational data, and conducting replication studies with larger, multi-center, or multi-regional samples to enhance generalizability. | 31-34 |
|  | 8. Limitations are correctly stated, but social desirability, selection bias, and the use of self-reported data only should be emphasized more strongly. Leadership, organizational support, and organizational environment variables should have been modeled. | We sincerely thank the reviewer for this valuable comment. We have revised the Limitations section to more strongly emphasize potential biases related to social desirability, selection bias, and the exclusive use of self-reported data. Additionally, we have acknowledged in the Discussion that leadership, organizational support, and environmental factors are important variables that should be incorporated in future predictive models. | 30-31 |
|  | 9. Clinical, educational, and management/policy-based recommendations are included, but the potential obstacles that may be encountered in implementing each recommendation or situations that may challenge existing practices at the country level have not been discussed. | We sincerely thank the reviewer for this insightful comment. We have revised the Implications section to include a discussion of potential obstacles and challenges that may be encountered when implementing clinical, educational, and management/policy recommendations, particularly in the context of local practices and healthcare systems. | 31-34 |
|  | 10. The discussion lacks a “paradigm shift in clinical practice” or scientific justification for policy recommendations; the recommendations remain largely at a universal level. | We sincerely thank the reviewer for this insightful comment. We have revised the Implications and Policy sections to provide a clear scientific justification for our recommendations, directly linking them to the study findings. In addition, we emphasized how implementing shared governance structures, structured case-review forums, and autonomy-enhancing policies has the potential to shift traditional hierarchical decision-making toward a more collaborative, evidence-informed approach, representing a paradigm shift in ICU clinical practice. | 23-29 |
|  | 11. The findings' “practice and policy” recommendations should be more concrete and detailed for institutions. | We sincerely thank the reviewer for this valuable comment. We have revised the Practice and Policy recommendations to make them more concrete and detailed, providing specific guidance for institutional implementation, including actionable steps, role assignments, and monitoring strategies. | 31-34 |
|  | 12. The implementation recommendations remain at a general level; more specific and practically applicable recommendations (e.g., “making interdisciplinary training mandatory”) could be presented. | We sincerely thank the reviewer for this valuable comment. We have revised the Implementation recommendations to include more specific and practically applicable actions, such as mandating interdisciplinary training, appointing clinical champions, piloting structured governance interventions, and defining measurable indicators for monitoring performance. | 31-34 |
|  | 13. The clinical and societal significance of the findings, their contribution to current practices, or how they could play a role in systemic improvement could be presented in greater detail. | We sincerely thank the reviewer for this insightful comment. We have revised the Discussion and conclusion sections to more clearly highlight the clinical and societal significance of our findings, their contribution to current ICU practices, and the potential role of enhancing autonomy and interprofessional collaboration in driving systemic improvements in patient care and safety. | 31-34 |
|  | 14. The distinction between the conclusion paragraph and the discussion is unclear; a strong closing section summarizing the importance of the main findings, their limitations, and the recommendations should be added. | We sincerely thank the reviewer for this constructive comment. We have revised the Conclusion section to provide a strong, independent closing paragraph that clearly summarizes the main findings, highlights key limitations, and presents practical and research recommendations. This revision ensures that the Conclusion is distinct from the Discussion while emphasizing the clinical, organizational, and systemic significance of the study. | 31-32 |
|  | 15. The reasons why the findings of the study may be only “partially” generalizable should be discussed more clearly; in particular, limitations in the sample, data collection method, and measurement tools may cast doubt on the reliability of the results. | We sincerely thank the reviewer for this comment. We have addressed all relevant points in the Limitations section, clearly discussing factors that may limit the generalizability of our findings, including sample characteristics, data collection methods, and the measurement tools used. | 23-29 |
|  | 16. Analysis and literature comparison should be structured according to hypotheses/objectives. | We sincerely thank the reviewer for this insightful comment. We have revised the Analysis and Discussion sections to structure the presentation of results and literature comparisons according to the study’s predefined hypotheses and objectives, ensuring a more precise and more systematic alignment between findings and research questions. | 23-29 |
|  | 17. The limitations section should be diversified with concrete examples (e.g., for decision-making performance, not only self-reporting but also objective data suggestions). | We sincerely thank the reviewer for this valuable comment. We have revised the Limitations section to include more concrete examples, such as suggesting the use of objective performance measures (e.g., direct observation or chart audits) alongside self-reported data, to provide a clearer understanding of potential biases and measurement limitations. | 30-31 |
|  | 18. Practical application and policy recommendations should include discussion of the specific context (country, institutional structure, culture), feasibility, and potential difficulties that may be encountered. | We sincerely thank the reviewer for this valuable comment. We have revised the Practical Application and Policy Recommendations sections to explicitly address the specific context of Iran, including institutional structures, cultural considerations, and feasibility issues. Potential challenges, such as limited staffing, resistance to organizational change, and resource constraints, have been discussed, and practical strategies to overcome these obstacles are now provided to ensure effective implementation. | 31-34 |
|  | 19. In the conclusion section, the impact of the study findings on the literature, nursing education, and management should be summarized and emphasized. | We sincerely thank the reviewer for this constructive comment. The Conclusion section has been revised to clearly summarize and emphasize the study's findings, highlighting their impact on the existing literature, nursing education, and management practices, and underscoring their significance for both clinical and organizational contexts. | 30-31 |
